# Childhood asthma and vitamin D deficiency in Turkey: is there cause and effect relationship between them?

**DOI:** 10.1186/1824-7288-39-78

**Published:** 2013-12-13

**Authors:** Metin Uysalol, Levent Cem Mutlu, Gamze Varol Saracoglu, Erkut Karasu, Savas Guzel, Semra Kayaoglu, Nedret Uzel

**Affiliations:** 1 Department of Pediatrics, Istanbul Faculty of Medicine, Istanbul University, Fatih/Capa, 34093 Istanbul, Turkey; 2 Department of Chest Diseases, Faculty of Medicine, Namık Kemal University, Tekirdag, Turkey; 3 Department of Public Health, Faculty of Medicine, Namık Kemal University, Tekirdag, Turkey; 4 Department of Pediatrics, Faculty of Medicine, Namık Kemal University, Tekirdag, Turkey; 5 Department of Biochemistry, Faculty of Medicine, Namık Kemal University, Tekirdag, Turkey; 6 Department of Family Medicine, Sisli Eftal Education and Training Hospital, Istanbul, Turkey

## Abstract

**Background:**

Epidemiological studies show that vitamin D deficiency and insufficiency are common worldwide and associated with many diseases including asthma. Our aim was to evaluate vitamin D insufficiency and its clinical consequences.

**Methods:**

This cross-sectional study was carried out on 170 children consisted of 85 who were asthmatic and 85 who were not, aged 2 to 14 years in Tekirdag, Turkey, from September 2009 to May 2010. Children’s basal serum D vitamin levels were determined, and their eating habits, vitamin D intake, exposure to sunlight and use of health services during the previous year were investigated. The severity of asthma and levels of asthma control were assessed according to the Global Initiative for Asthma guidelines.

**Results:**

The difference between mean vitamin D levels in the asthmatic group (mean +/- SD) 16.6 +/- 8.5 ng/mL and the healthy control group (mean +/- SD) 28.2 +/- 19.5 ng/mL was found to be statistically significant (p < 0.001). Children in the asthma group had less exposure to sunlight and ate a diet less rich in vitamin D (p < 0.001). A significant difference was observed between the groups regarding the frequency of respiratory tract infections leading to emergency unit admissions and number of hospitalizations (p < 0.001). It was also shown that a decrease in vitamin D level increased the severity of asthma (p < 0.001) and decreased the frequency of controlled asthma (p = 0.010).

**Conclusion:**

This study has demonstrated the correlation between plasma 25 (OH) D levels and childhood asthma. Evidently, this relationship being influenced by multiple factors other than vitamin D, further studies should be conducted to explore the interrelation between all such factors.

## Background

Despite adequate exposure to sunlight and satisfactory diet, vitamin D deficiency is still widespread in many countries [1]. A study conducted in Turkey showed that plasma vitamin D levels were lower than 20 ng/ml, the vitamin D deficiency ratio was 25% and vitamin D insufficiency was 15% [2]. Vitamin D is obtained via exposure to sunlight or from foods that naturally contain vitamin D. Other sources include vitamin D rich foods or vitamin D enriched foods and vitamin D oral intake. In addition, vitamin D level is determined by sex, age, area of residence, skin pigmentation, obesity and dietary habits [3-5]. In some studies, it is stated that asthmatic children were allowed to spent less time outdoors and feed less with vitamin D enriched foods with the belief that seasonal weather changes and vitamin D enriched foods triggered asthma attacks [6-8].

Various studies have investigated the difference in vitamin D levels between asthmatic and non-asthmatic children [6,8-12] and the effects of vitamin D deficiency in children on the severity and control of asthma [6,12-14]. Some authors claim that vitamin D deficiency is associated with the risk of increasing respiratory infections [15], leading to exacerbation of asthma and subsequent frequent hospitalizations [6]. It is reported that relatively less incidence of asthma exacerbation is observed in asthmatic children taking vitamin D supplements [16]. Various review articles have suggested a strong link between vitamin D levels and asthma severity, although the causality has not been proven [7,17,18]. The high prevalence of vitamin D insufficiency observed in dark-skinned people of certain races living in the Northern hemisphere may suggest a relationship between vitamin D level and asthma severity [6,11,12,14]. It is reported that increasing vitamin D level by oral intake decreases asthma-related morbidity in both healthy and asthmatic children with vitamin D deficiency [13]. Although a few clinical studies indicate that vitamin D may be useful in reducing the incidence of respiratory diseases with wheezing and of asthma resulting from viral respiratory infections, some other studies have found contrary results, noting that significant differences existed in the evaluation of vitamin D intake, timing of vitamin D sampling, the values of the vitamin D threshold used to define deficiency, and results of respiratory tests. Thus, recommendations of vitamin D treatment for asthma cannot be made, as the observational studies have yielded unclear indicators [17,18].

Accordingly, further studies are needed to reveal such causal relationship as may exist and to suggest treatments. In this study, we assumed that the vitamin D level was lower in asthmatic children than in non-asthmatic children and analyzed the prevalence of vitamin D deficiency in these groups. Considering that factors such as exposure to sunlight, vitamin D supplement and diet also affect vitamin D levels in children, we further assumed that there would be a difference in the number of respiratory infections, emergency admissions and hospitalizations recorded among patients according to their vitamin D levels. We attempted to verify whether the vitamin D level has any effect in determining the severity and control of asthma. Our study analyzed the relationship between vitamin D deficiency and asthma in 85 light-skinned asthmatic and 85 light-skinned non-asthmatic children living in Tekirdag.

## Methods

This cross-sectional study was carried out on 170 children aged 2 to 14 years, referred to Paediatric Outpatient Clinics of Namık Kemal University Research and Teaching Hospital, Tekirdag, Turkey, from September 2009 to May 2010. All the families filled out a written consent form. The study fulfilled the Helsinki criteria and ethical consent was obtained from the Namık Kemal University Ethical Committee.

The subjects included 85 asthmatic patients, diagnosed according to the Global Initiative for Asthma (GINA) criteria: 1) a physician’s diagnosis of asthma, 2) symptoms of recurrent (i.e. more than two) episodes of wheezing, cough, shortness of breath, or a combination of these, 3) documented reversibility with bronchodilators, and 4) symptoms of and/or use of medication for asthma in the previous six months [19]. The control group included 85 children between the ages of 2 and 14 years who came to the Social Paediatric Outpatient Clinic at the same hospital and who were previously healthy and did not suffer from any acute or chronic illnesses such as allergy, asthma or other respiratory tract infections. For both the study and control groups, children were chosen from similar environmental backgrounds, from the same local population and from similar sites with the same epidemiological characteristics. The study and the control group were chosen simultaneously so as not to change the biochemical parameters due to weather conditions. Those receiving vitamin D therapy subsequent to any disease, anti-epileptic therapy or long-term steroid treatment besides asthma treatment were excluded from the study, as were those with chronic diseases other than asthma, such as lung disease, renal disease, liver disease and endocrine disease.

Patients’ detailed personal medical histories were recorded and the physician conducting the study performed a thorough physical examination and analysis of information. Socio-demographic characteristics such as age, sex and place of residence and morphometric measurements such as height and weight were recorded during meetings with the children’s family. The weight/height^2^ formula was used to calculate each child’s Body Mass Index (BMI). The child’s daily mean period of exposure to sunlight was determined. Exposure of 10 minutes between the hours of 10:00 and 15:00 was thought to provide approximately 2000–3000 IU of Vitamin D [18]. Vitamin D intake was estimated using a food frequency questionnaire, and questions were asked about children’s eating habits of vitamin D-rich and vitamin D enriched foods. Foods rich in vitamin D included salmon (cooked), 100 grams 360 IU; mackerel (cooked) 100 grams 345 IU; sardine (canned), 50 grams 250 IU; tuna fish (canned), 100 grams 235 IU; 60 grams of whole egg 20 IU; beef liver, cooked 100 grams 15 IU; one glass of milk 100 IU; and one spoonful of fish oil 400 IU. An intake of 400 IU vitamin D was considered to be a vitamin D-rich diet [20]. Respondents were asked about their consumption of vitamin D fortified bread and milk. If they consumed such foods every day, it was considered that they were having a vitamin D rich-diet. Daily intake of 400 IU of vitamin D; either by vitamin D only or with multivitamins was accepted as adequate vitamin D supplement for children. Questions were asked about the number of respiratory tract diseases subjects had experienced in the previous year, the number of emergency admissions and hospitalizations and the number of asthma attacks. Asthma was classified as intermittent, mild persistent, moderate persistent and severe persistent, according to GINA guidelines. Control of asthma was classified as uncontrolled, partially controlled and controlled, again according to GINA guidelines [19].

Appropriate laboratory tests were conducted to establish white blood cell count, eosinophil count, plasma IgE level, plasma vitamin D level, plasma calcium (Ca), phosphorus (P), alkaline phosphatase (ALP) and parathormone (PTH). Chest X-rays were also carried out to eliminate cases of disease other than asthma. Blood was collected in a serum separator tube (Vacutainer; Becton Dickinson, France) and allowed to stand for 30 minutes at room temperature to ensure full clotting. All samples were subsequently centrifuged at 3000 × g for 5 minutes, and the supernatant was aliquoted and analysed. Whole blood samples were collected in EDTA (K3) tubes (Becton Dickinson, France). Plasma 25-hydroxyvitamin D3: 25-hydroxy D is a reliable measure of plasma vitamin D level [21]. The plasma concentration of vitamin D was assayed using an electrochemiluminescence immunoassay on the Cobas E 411 auto analyser (Roche Diagnostics GmbH, Mannheim, Germany). This assay for vitamin D is sensitive down to a concentration of 4.0 ng/mL. The coefficient of variation (CV) using quality control samples was 4%. Plasma intact PTH level assay: intact PTH was measured using an electrochemiluminescence immunoassay on the Cobas E 411 auto analyser. Its reference value in our laboratory was 15–65 pg/ml, and intra- and interassay CVs were 2.7 and 6.5%, respectively. Plasma total IgE and peripheral blood eosinophil count: plasma levels of total IgE were determined using the electrochemiluminescence immunoassay (Cobas E 411). The lower detection limit of the assay was 0.1 IU/mL. The coefficient of variation using quality control samples was 3.7%. The intra- and interassay CVs were 4.1 and 5.1%, respectively. Peripheral leukocyte analyses included total leukocyte counts and percentages of eosinophil, using an automated cell counter (XT-2100i Hematology Alpha Transportation System; Sysmex, Kobe, Japan). The absolute count of eosinophil was calculated as the product of its percentage and the total leukocyte count. Serum total calcium, inorganic phosphorus and alkaline phosphatase were measured using an automated analyser (Cobas C 311, Roche Diagnostics). Serum calcium was adjusted for albumin; corrected serum calcium = (serum calcium [measured] + 0.8 * [4- serum albumin]).

The study was designed to include 170 participants (85 experimental and 85 control participants). The response within each study group was normally distributed with a standard deviation of 0.5 in order to reject the null hypothesis with a power of 1.00 and a risk of type I error of 5%; Descriptive analysis for the socio-demographic aspects of the study and the control group was performed using three levels of vitamin D, namely ≥ 30 ng/ml, 20–29 ng/ml and <30 ng/ml. P values were calculated by using two-sided t-tests for continuous predictors with equal variance. The Spearman rank correlation coefficient was used when the variables were continuous and the χ2 test was used when they were categorical. All the statistical analyses were evaluated within a 95% two-sided confidence interval (CI).

## Results

Children included in this study were asthmatic patients who were all light-skinned with normal BMI (< 85th percentile), and their mean age and sex distribution were similar to those of healthy controls (p > 0.05). Their mean serum vitamin D levels were significantly higher than those of healthy controls (p < 0.001). The mean serum Ca and PTH levels were significantly higher in healthy controls compared to asthma patients (p = 0.04, p = 0.004, respectively). The mean plasma eosinophil level and serum IgE levels were significantly higher in asthmatic patients than in healthy controls (p < 0.001). On the other hand, the mean serum P and ALP levels did not differ between the two groups (p = 0.995, p = 0.996, respectively) (Table 1).

**Table 1:** The comparison of biochemical parameters related to asthma between the study and the control group

**Characteristics**	**Asthma group**	**Control group**	**p**
	**(n = 85)**	**(n = 85)**	
mean ± SD			
**Age (years)**	6.2 ± 2.4	6.6 ± 2.8	>0.05
**BMI (kg/m**^**2**^**)**	17.7 ± 3.2	17.3 ± 3.0	>0.05
**Vitamin D (ng/ml)**	16.6 ± 8.5	28.2 ± 19.5	<0.001
**Total IgE (IU/ml)**	236.4 ± 323.2	65.4 ± 102.9	<0.001
**Eosinophil count (cell/mm**^**3 **^**)**	5.8 ± 2.3	3.4 ± 2.8	<0.001
**Calcium (mmol/L)**	9.6 ± 0.6	9.8 ± 0.6	0.04
**Phosphorus (mmol/L)**	5.1 ± 0.7	5.2 ± 0.7	0.995
**Alkaline phosphatase (U/L )**	197.7 ± 86.8	192.7 ± 78.3	0.996
**Parathyroid hormone (mmol/L)**	37.2 ± 17.0	29.9 ± 16.2	0.004

SD: Standard Deviation, p: student’s t test.

**Figure 1:**
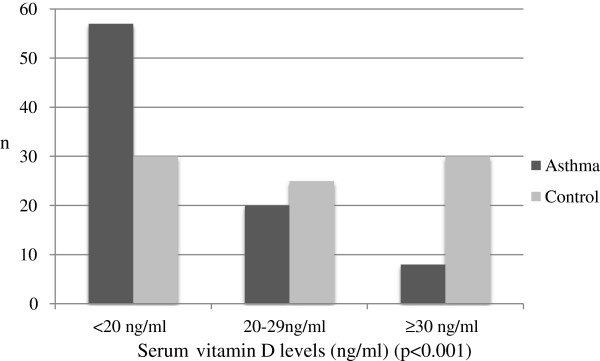
Distribution of serum vitamin D levels in Turkish children with asthma and control groups.

Figure 1 shows the distribution of serum vitamin D levels in the asthma and healthy control groups. Serum vitamin D levels of ≥ 30 ng/ml (considered sufficient) were found in 78.9% of 85 healthy children (n = 30), compared with only 21.1% of 85 asthmatic children (n = 8). Serum vitamin D levels of < 30 ng/ml (considered insufficient or deficient) were found in 67.7% of control group (n = 55), compared with 90.6% (n = 77) of the asthmatic group. Serum vitamin D levels of 20–29 ng/ml (considered insufficient) were found in 67% of asthmatic children (n = 20) and in 35.3% (n = 25) of the control group. Serum vitamin D levels of < 20 ng/ml (considered deficient) were found in 29.4% of asthmatic children (n = 57) and in 23.5% of the healthy control group (n = 30). There was a significant difference between the vitamin D levels in children and classification of serum vitamin D levels (p < 0.001) (Figure 1).

The parameters affecting vitamin D level, such as adequate vitamin D intake, supplemented/unsupplemented vitamin D intake, duration of the children’s exposure to sunlight and place of residence were investigated in both asthmatic and control groups. Asthmatic children had less exposure to sunlight (p < 0.001) and followed a diet less rich in vitamin D (p < 0.001) than the healthy children. There was no significant difference between the asthmatic and control groups with respect to vitamin D intake during breastfeeding (p = 0.61) or living in a rural or an urban area (p = 0.501). While there was no significant difference in exposure to sunlight and level of vitamin D within the asthmatic group (p = 0.07), there were significant differences within the control group (p = 0.001). There was a significant difference between the vitamin D level and the intake of a vitamin D rich diet within the asthmatic and control groups (p = 0.04, p = 0.013, respectively). There was no significant difference within the asthmatic and control groups with respect to vitamin D intake during breastfeeding (p = 0.141, p = 0.302, respectively), current vitamin D level and residence in a rural or an urban area (p = 0.342, p = 0.245, respectively) (Table 2).

**Table 2:** Duration of sun exposure, eating habits, vitamin D supplement during breast feeding and place of residence in the asthmatic and control group children

	**Groups (n = 170)**					
	**Asthma group (n = 85)**			**Control group (n = 85)**		
**Characteristics**	**<20 ng/ml n (%)**	**20-29 ng/ml n (%)**	**≥30 ng/ml n (%)**	**<20 ng/ml n (%)**	**20-29 ng/ml n (%)**	**≥30 ng/ml n (%)**
**Duration of sun exposure**				Groups (n = 170)		
**0-15 min**	20 (76.9)	2 (7.7)	4 (15.4)	1 (50.0)	0 (0.0)	1 (50.0)
**16-30 min**	23 (71.9)	7 (21.9)	2 (6.2)	7 (46.7)	1 (6.7)	7 (46.7)
**31-60 min**	8 (42.1)	9 (47.4)	2 (10.5)	14 (31.1)	17 (37.8)	14 (31.1))
**61 min and +**	6 (75.0)	2 (25.0)	0 (0.0)	8 (34.8)	7 (30.4)	8 (34.8)
**p**	0.07			0.001		
**Eating habits**						
**D vitamin insufficient**	30 (76.9)	5 (12.8)	4 (10.3)	13 (61.9)	4 (19.0)	4 (19.0)
**D vitamin sufficient**	27 (58.7)	15 (32.6)	4 (8.7)	17 (26.6)	21 (32.8)	26 (40.6)
**p**	0.04			0.013		
**Vitamin D supplement**						
**No intake**	44 (73.3)	15 (32.6)	4 (8.7)	24 (32.0)	23 (30.7)	28 (37.3)
**Intake**	13 (52.0)	12 (20.0)	4 (6.7)	6 (60.0)	2 (20.0)	2 (20.0)
**p**	0.141			0.302		
**Place of residence**						
**Urban**	41 (70.7)	11 (19.0)	6 (10.3)	19 (30.6)	18 (29.0)	25 (40.3)
**Rural**	16 (59.3)	9 (13.3)	2 (7.4)	11 (47.8)	7 (30.4)	2 (21.7)
**p**		0.342			0.245	

The frequency of respiratory infections in the previous year and the use of health services in relation to serum vitamin D levels in the asthmatic and control group children were evaluated. Considering the frequency of respiratory tract infections (RTI), seen in the previous year; it was noted that the vitamin D level decreased with increased RTI frequency in both the asthmatic group (r = −0.55; p < 0.01) and the control group (r = −0.47; p < 0.01).

**Figure 2:**
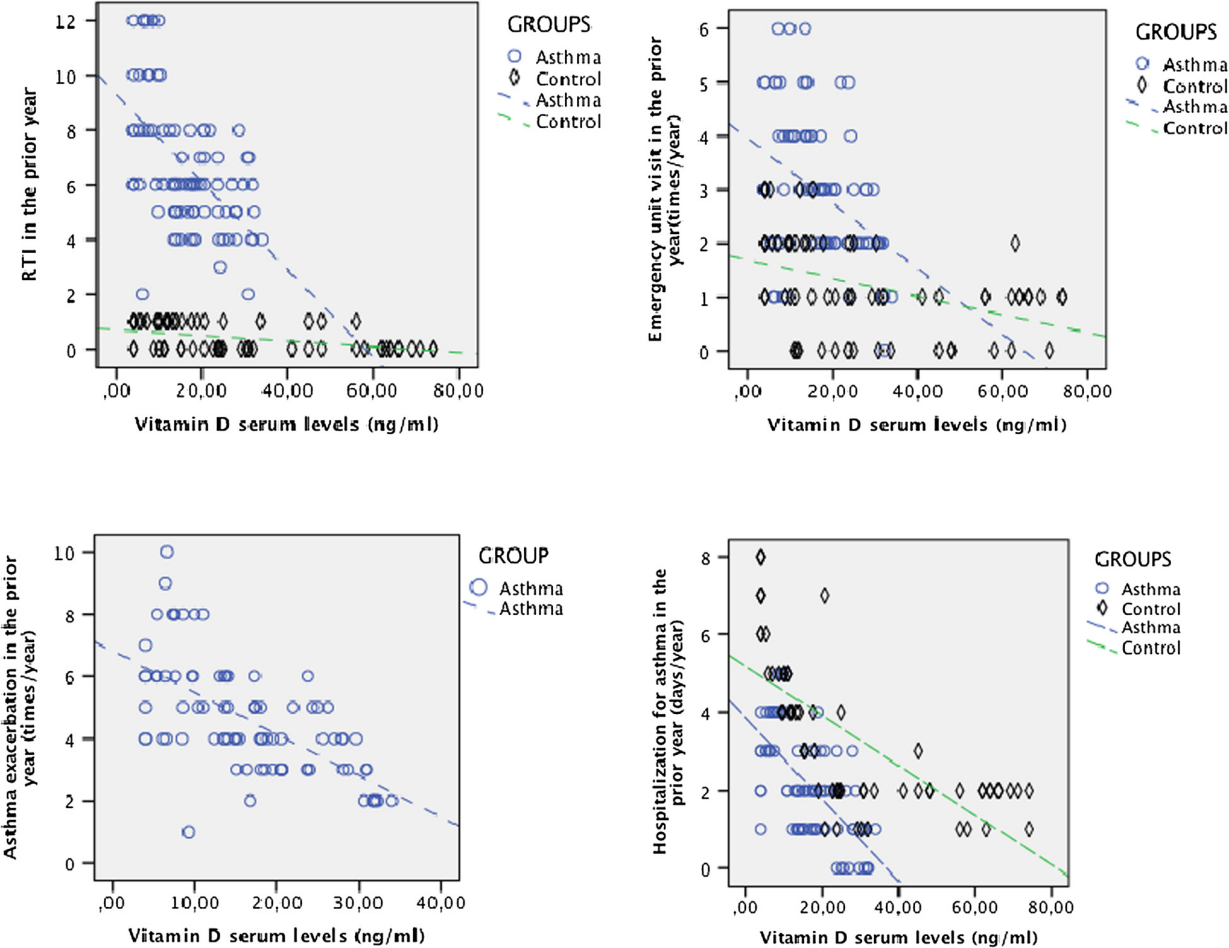
The distribution of number of previous year respiratory tract infections, emergency department admissions, and hospitalizations in asthmatic and control group children.

A negative correlation was observed between vitamin D decrease and emergency unit (EU) admissions both in the asthmatic and the control group (asthma r = −0.39; p < 0.01; control group r = −0.43; p < 0.01). The hospitalization ratios increased as vitamin D levels decreased both in the asthmatic (r = −0.65; p < 0.01) and the control groups (r = −0.71; p < 0.01). Asthma attacks in the previous year increased with a decrease in vitamin D level (r = −0.63; p < 0.01). There was a negative correlation between vitamin D levels and emergency admissions, hospitalization ratios and RTI frequency in both the asthmatic and the control groups. There was a negative correlation between vitamin D levels and frequent asthma attacks in the asthmatic group (Figure 2).

**Figure 3:**
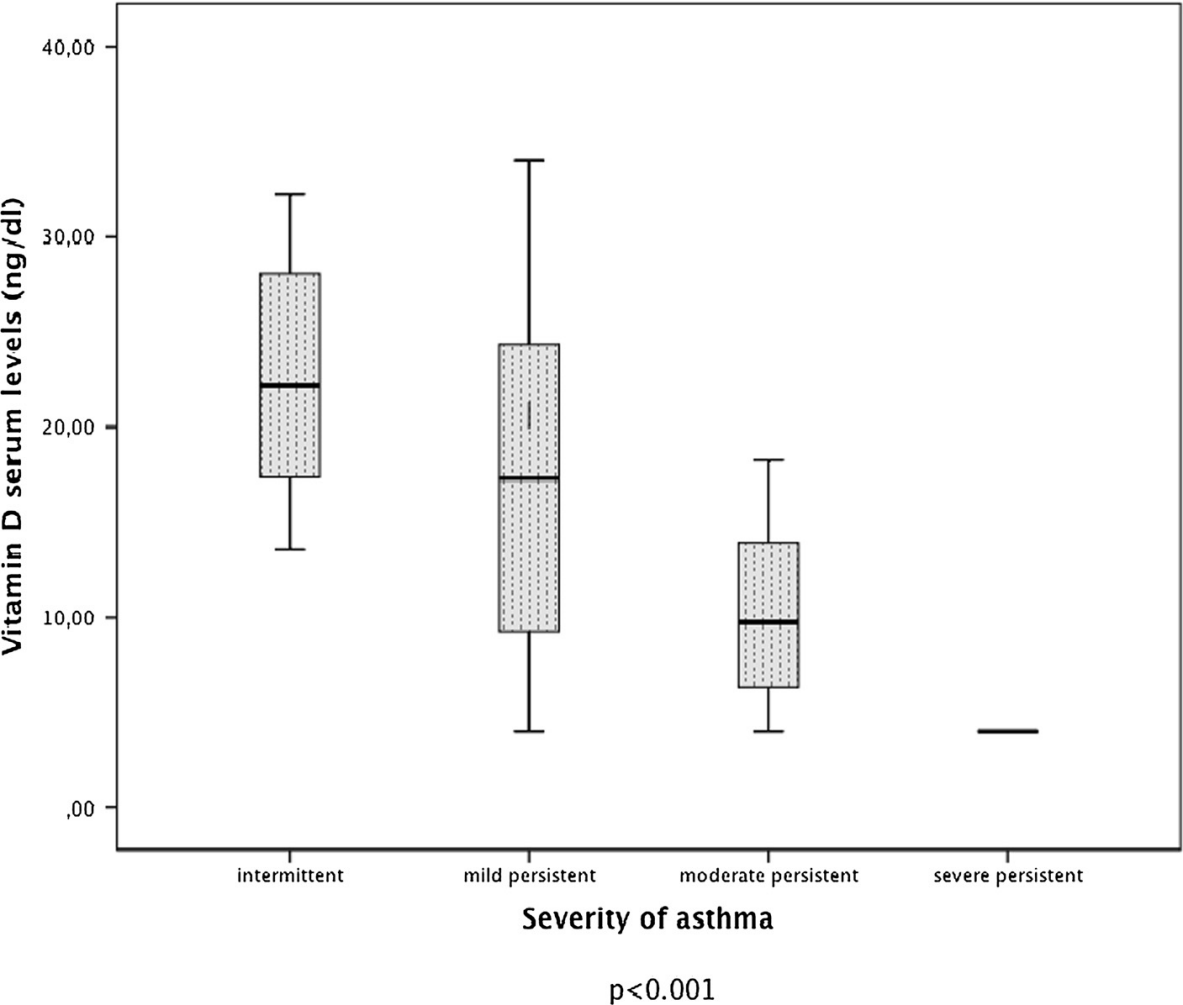
The box plot of serum vitamin D levels by severity.

**Figure 4:**
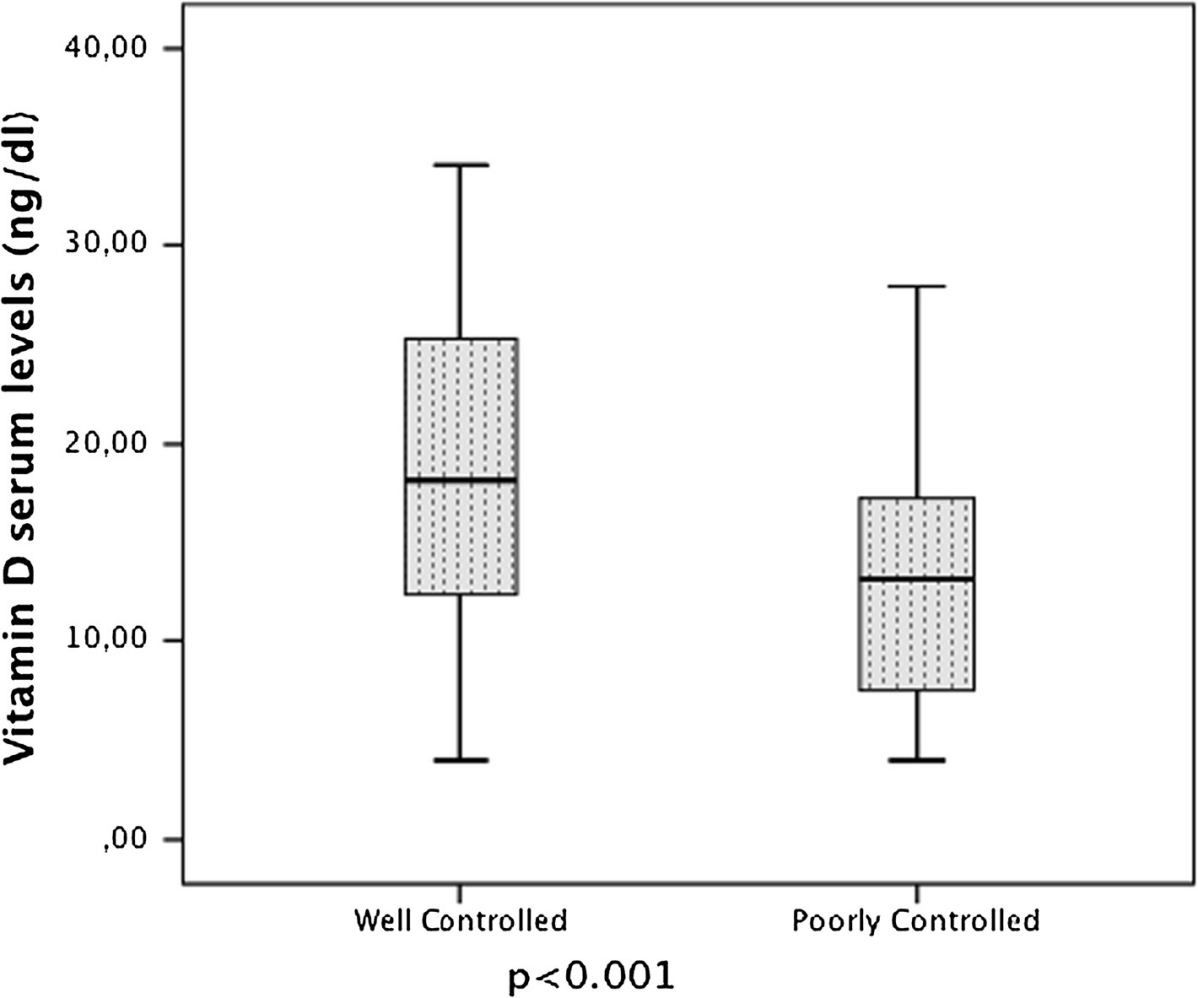
The box plot of serum vitamin D levels in well Controlled and poorly Controlled, asthmatic patients.

Vitamin D serum levels in children were grouped according to the GINA classification of asthma severity, as shown in Figure 3. In the asthmatic group, vitamin D mean ± SD values were, respectively, intermittent: 22.46 ± 6.03; mild persistent: 17.70 ± 9.08; moderate persistent: 10.39 ± 4.84; and severe persistent: 4.00 ± 0.0. As the asthma severity increased, the vitamin D levels decreased. There was a significant difference between the vitamin D levels in children and classification of asthma severity (p < 0.001). The distribution of vitamin D serum levels in the children grouped according to the GINA classification of asthma control is shown in Figure 4; the vitamin D values were controlled: 18.81 ± 8.74 and poorly controlled: 13.09 ± 6.95, respectively. Within the asthmatic group, the levels of vitamin D and severity of disease and degree of control were compared and significant differences were found. As the vitamin D level decreased, the severity of asthma increased (p < 0.001) and the frequency of controlled asthma disease decreased (p = 0.010).

## Discussion

In our study, vitamin D insufficiency and deficiency were found to be significantly higher in asthmatic children than in the control group, and these values were higher than values reported in other studies [6,8,11,12,14]. Vitamin D levels are classified as sufficient (≥ 30 ng/ml), insufficient (20–29 ng/mL) and deficient (< 20 ng/ml); and in our study these accounted for 9.5%, 20%, and 67% of the subjects (Figure 1), respectively. Studies conducted in Italy on children with a mean age close to those we studied, [14] and in the Middle East [10] showed similar results, whereas the percentages found in a study in Iran were respectively higher (84%, 12% and 4%) [9]. Similar studies from another countries revealed variable results that can be explained by ethnicity, skin colour and maternal supplementation during pregnancy [6,8,11-13,22,23]. Examples of these discrepancies include residence in an urban environment and dark-skin.

The primary source of vitamin D is from the skin’s production upon exposure to sunlight; secondary sources are eating vitamin D-rich and vitamin D enriched foods [12]. Our study showed that asthmatic children had less exposure to sunlight compared to control group (p < 0.001). This is explained by the fact that families wanted to keep their children at home for fear of an asthma attack if they went outdoors or engaged in physical activities. Bener et al. found similar results (p = 0.006) [10]. In another study, no significant difference was found between vitamin D levels and their asthmatic children’s approximate three hours of exposure to sunlight per day (p = 0.49); however, the children in that study were dark-skinned and lived in an urban environment [22]. In our study, finding no significant difference between exposure to sunlight and vitamin D levels led us to think that there were other factors and mechanisms determining serum vitamin D levels in asthmatic patients. A vitamin D insufficient diet was found significantly more often in the asthmatic group than in the healthy control group (p < 0.001). The fear of allergic reaction against diet enriched with vitamin D might be reason in this area.

In Turkey, vitamin D-rich foods are widely available but few people are familiar with this fact. We conclude that families need to be better informed about nutrition. Our findings were similar to a study from Puerto Rico, which examined the vitamin D levels in “high dietary intake of vitamin D” group and an asthmatic group [11].

Vitamin D supplements, which were given to the children during breastfeeding, did not make any significant difference in the serum vitamin D levels of the asthmatic and control groups. As a similar result, Bener et al. reported that there was no significant difference (p = 0.561) between asthmatic and control groups when the vitamin D supplements were given to the children during breastfeeding [10].

It is considered to be appropriate to increase the dosage of vitamin D and duration of vitamin D intake in Turkey (current vitamin D supplement level is 400 IU), according to the latest report of The Institute of Medicine of the National Academies in the United States of America. The vitamin D currently added to foods and the use of vitamin D as a replacement therapy have been shown not to eliminate vitamin D insufficiency [24] or vitamin D deficiency [25,26]. The Institute of Medicine of the National Academies in the United States of America, in its latest report of 2011, recommended increasing the nutritional dose of vitamin D from 400 to 600 IU for children older than one year of age [27]. We concur that it is necessary to increase the vitamin D dose, the period of exposure to sunlight, and the time of exposure to sunlight (preferably around midday). Equally, we must consider protective clothing, the use of sun blocks and the adverse effects of UVB in certain geographical regions. Further, we think that the relationship between vitamin D deficiency and asthma should be reinvestigated, taking into consideration the social eating habits, the dose and the duration of vitamin D supplementation.

When analysing the RTIs of the previous year, we observed that as vitamin D levels decreased, RTI frequency increased in both the asthmatic and the control groups, and there were frequent incidences of RTI in asthmatic patients with low vitamin D levels [15], leading to an increase in the severity of the asthma attack [6]. The number of hospital admissions due to respiratory complaints increased as serum vitamin D decreased in the asthmatic group. Although some studies had similar results to ours [6,12,14], a study by Alyasin et al. found no relationship between vitamin D levels and hospitalizations [9]. A negative correlation was found between serum vitamin D level and asthma attacks, which increased significantly in frequency as serum vitamin D decreased. Recent studies showed that vitamin D deficiency led to an increase in the risk of asthma attack [6,14,16]. Some studies indicated an inverse relationship between vitamin D levels and use of health services [6,11,12], while others showed no such relationship [9,22].

In our study, the increase in IgE and eosinophil count in asthma patients (Table 1), RTI, asthma attacks, EU admissions and number of hospitalizations for treatment were observed in relation to vitamin D levels. This led us to believe that vitamin D deficiency increased the severity of asthma, complicating control of the disease. The relationship between vitamin D deficiency and severity of asthma has been investigated and it was reported that the severity of asthma increased with vitamin D deficiency [6,8,9,11,28]. The relationship between vitamin D concentration and control of asthma was investigated in some studies conducted generally in North American and Costa Rican populations. In these studies, the subjects belonged to certain ethnic groups and were dark-skinned, urban schoolchildren, and they reported that D vitamin insufficiency was associated with less time spent outdoors, increased total IgE concentrations, eosinophil counts, airway hyper-responsiveness, and increased symptoms and exacerbations [11,28]. We should also bear in mind that there could be a decrease in vitamin D levels in children who go outdoors less, owing to asthma attack exacerbation and poor control of the illness.

Our study had some limitations. First, the sample size was relatively small (n = 170). A larger sample size would have increased our statistical power to detect associations. As asthma diagnosis can only be achieved after the age of six due to difficulties with applying spirometers and measurement of exhaled nitric oxide, our diagnoses mostly rely on physicians’ experiences [29,30]. As with most other studies investigating the role of vitamin D in asthma, our design was cross-sectional, thus limiting our ability to establish a causal link between vitamin D and asthma morbidity. Future clinical trials are necessary to determine if vitamin D truly has effects upon asthma as suggested by the observational literature.

## Conclusions

In summary, in our study, we tried to clarify the role of vitamin D in asthma. We found that vitamin D levels were considerably lower in children with asthma than in healthy children. Exposure to sunlight and a diet rich in vitamin D increased the vitamin D levels in both asthmatic and healthy children. The children with asthma and healthy children with low vitamin D levels had frequent RTIs, leading to more EU admissions and to more use of health services owing to more hospitalizations. A higher frequency of asthma attacks, more severe asthmatic episodes and greater difficulty in asthma control were observed in asthmatic children with low vitamin D levels. We found a relationship between serum vitamin D levels and asthma; however, this relationship could be influenced by multiple factors. Thus, there remains a need to investigate further the relationship between asthma and vitamin D in a multidisciplinary fashion.

## Abbreviations

GINA: Global initiative for asthma; BMI: Body mass index; Ca: Calcium; P: Phosphorus; ALP: Alkaline phosphatase; PTH: Parathormone; CV: Coefficient of variation; CI: Confidence interval; RTI: Respiratory tract infections; EU: Emergency unit.

## Competing interests

The authors declare that they have no competing interests.

## Authors’ contributions

MU conceived of the study, and participated in its design and coordination and drafted the manuscript. LCM participated in the design of the study, and collection and acquisition of data. EK helped to collection and acquisition of data. SG carried out biochemical and hormonal analysis. GVS performed the statistical analysis. SK participated in the design of the study. NU helped to draft the manuscript. All of the authors read and approved the final version of the manuscript.
